# Prediction of Behaviour of Thin-Walled DED-Processed Structure: Experimental-Numerical Approach

**DOI:** 10.3390/ma15030806

**Published:** 2022-01-21

**Authors:** Miroslav Urbánek, Josef Hodek, Daniel Melzer, Martina Koukolíková, Antonín Prantl, Jaroslav Vavřík, Michal Brázda, Petr Martínek, Sylwia Rzepa, Jan Džugan

**Affiliations:** COMTES FHT a.s., Prumyslova 995, 33441 Dobrany, Czech Republic; josef.hodek@comtesfht.cz (J.H.); daniel.melzer@comtesfht.cz (D.M.); martina.koukolikova@comtesfht.cz (M.K.); antonin.prantl@comtesfht.cz (A.P.); jaroslav.vavrik@comtesfht.cz (J.V.); michal.brazda@comtesfht.cz (M.B.); petr.martinek@comtesfht.cz (P.M.); sylwia.rzepa@comtesfht.cz (S.R.); jan.dzugan@comtesfht.cz (J.D.)

**Keywords:** additive manufacturing, DED process, structure evaluation, ductile damage, FEM

## Abstract

Additive manufacturing (AM) becomes a more and more standard process in different fields of industry. There is still only limited knowledge of the relationship between measured material data and the overall behaviour of directed energy deposition (DED)-processed complex structures. The understanding of the structural performance, including flow curves and local damage properties of additively manufactured parts by DED, becomes increasingly important. DED can be used for creating functional surfaces, component repairing using multiple powder feeders, and creating a heterogeneous structure with defined chemical composition. For thin parts that are used with the as-deposited surface, this evaluation is even highly crucial. The main goal of the study was to predict the behaviour of thin-walled structures manufactured by the DED process under static loading by finite element analysis (FEA). Moreover, in this study, the mechanical performance of partly machined and fully machined miniaturized samples produced from the structure was compared. The structure studied in this research resembles a honeycomb shape made of austenitic stainless steel AISI 316L, which is characterized by high strength and ductility. The uncoupled damage models based on a hybrid experimental-numerical approach were used. The microstructure and hardness were examined to comprehend the structural behaviour.

## 1. Introduction

Additive manufacturing (AM) as an innovative and versatile technology is utilized in different sectors, such as the automotive, aerospace, and defense industries. In medicine, it is used to allow the creation the structures of complicated geometry based on topological optimization. Powder bed fusion (PBF) and directed energy deposition (DED) are two main systems for metal powder processing. The difference between these two processes is related to the material handling. In the PBF process, the layer of new powder material is selectively melted, while in DED the powder or wire is injected into the melt pool.

The DED can be used for the repair and remanufacture of the components during their lifetime, as discussed by Saboori et al. [1,2]. In a critical review, Feenstra et al. focused on the possibility of deposition of multimaterial builds, which is a unique advantage of DED systems [3]. They pointed to the problems that can occur during the process, such as the formation of intermetallic phases, cracking and delamination as well as difficulties related to process control. Therefore, the development of advanced numerical simulation tools and a broad-material database is necessary for better understanding of the DED deposition parameters’ effect on the material properties and microstructure [3]. The authors also mentioned that DED systems have a disadvantage of lower powder efficiency (approximately 30%) in comparison to other powder-processing technologies such as PBF, where efficiency can reach even 90% [4]. Jackson et al. compared the microstructure and mechanical properties of the DED-processed parts made of 316L deposited using gas-atomized to those of mechanically-generated feedstock, which is a potentially lower-cost feedstock alternative. The authors concluded that both variants of build exhibited comparable surface quality, microstructure and mechanical properties except hardness and ultimate tensile strength, which reached higher values in the case of mechanically-generated feedstock. Further research needs to be conducted on the effect of processing parameters and interaction of the feedstock material with machining tools [5]. In another critical review, Kok et al. [6] addressed a broad range of problems related to the proper and efficient use of metal additive manufacturing, mainly for load-bearing components. However, the study is focused mainly on titanium alloys, but no comments are given on stainless steels. According to Kiran et al., the DED process can be compared to multi-track welding, in which the repeated thermal cycles (heating and rapid cooling) directly influence structural integrity of the build, the generation of residual stresses and part distortion [7]. A broad discussion of the 316L material manufactured by the DED process concerning porosity, density, and microstructure was also carried by Tan et al. [8], Saboori et al. [9], and Bevan et al. [10]. A comparison between rolled and 3D-printed stainless steel produced by selective laser melting was performed by Natali et al. [11]. Also, many investigations have been performed in the field of fatigue behavior; for example, Gordon [12]. Nevertheless, the ductile damage under complex stress states including static and dynamic conditions has not been tackled for the DED process yet.

Currently, most of the studies in the field of additive manufacturing are dedicated to PBF. The existing studies, dealing with the DED process, concentrate mainly on the technological and processing aspects, the microstructure, and conventional mechanical properties characterization. The effect of processing parameters on the microstructure and tensile properties of austenitic steels made by DED was studied by Wang et al. [13]. In this study, the mechanical behaviour of DED-processed austenitic steels (including 316L) was compared to the performance of conventionally produced material. Based on the results of a tensile test, it was assumed that the DED-processed austenitic steel is characterized by a higher yield and tensile strength as compared to the same steel produced in a standard way. In addition, no clear anisotropy was identified [13]. Aversa et al. studied the effect of process parameters on the microstructure and mechanical performance of DED-ed parts made of stainless steel 316L. The results suggest that the deposition parameters affect the cell size of the cellular structure created during the process. In addition, the parts manufactured applying lower power value were distinguished by higher strength and higher porosity [14].

In this study, the ductile damage models are based on a phenomenological approach as presented by Bai, Bao, Wierzbicki [15], and Gu, Mohr [16] rather than on the continuum damage models of Gurson-Tvergard et al. or Lamaitre et al. [17]. This approach has been used for DED manufactured 316L steel by Azinpour et al. [18].

According to Nalli et al. [19], the lode angle parameter including ductile damage models are suitable characteristics in the assessment of material mechanical behaviour. A probabilistic fracture mechanics was studied by Tancogne-Dejean [20] on titanium alloys fabricated by additive manufacturing. The extensive testing programme was performed leading to the formulation of a non-associated plasticity model in a combination with the Hosford-Coulomb fracture model. The usage of this approach was motivated by a relatively high scatter of measured values as compared to conventionally produced titanium sheets. Nevertheless, this research work did not include any comparison of evaluated results with the behaviour of real components. The material model evaluation was based on an experimental-numerical approach obtaining the critical values of stress state and strain using the finite element model (FEM) of testing samples. To the best of our knowledge, the full combination of DED-processed material characterization and structure evaluation has not been published by this author yet.

There are numerous studies concerning the mechanical performance of additively manufactured parts made of austenitic stainless steels like the crushing of thin-walled circular tubes with internal grooves made by Yang et al. [21], the structural performance of square columns by Buchanan [22], the mechanical behaviour of honeycomb structures by Anandan et al. [23], Zahaira et al. [24] and the square and round tubular sections by Yan et al. [25]. All of these structures were fabricated by PBF. Considering the build geometry proposed in this research, it was important to get the information and experience from works dealing with thin-wall structures made by DED or similar processes like Cunningham [26], Wu [27], and Jin [28]. The mechanical properties of thin-walled honeycomb structures made of 316L stainless steel were investigated by Feldhausen et al. The components were produced by hybrid manufacturing, which allowed a decreasing of total porosity and the obtaining of consistent and repeatable tensile characteristics. However, the controlling of a part distortion can be a strong limitation of this method [29]. According to the study of Baranowski et al., a honeycomb geometry can be a unit cell of a network of interconnected walls which are periodically repeated within the component. Even though these components are characterized by a reduced relative density, they have a high strength, so can be applied as constructional materials [30]. Furthermore, Antolak-Dudka et al. investigated the mechanical behavior of the honeycomb structures by compression test under quasi-static and dynamic loading conditions. The results revealed that with increasing relative density of the component, a strain rate sensitivity also increases [31].

The goal of this study was a prediction of the behaviour of a DED-processed part under loading by numerical simulation using a material plasticity model including damage initiation and fracture. The main simulation input was a material model set up based on the results of mechanical tests performed using miniaturized samples extracted from the part. Finally, an experimental compression test was performed for the verification of the behaviour of the part under loading.

**Figure 1:**
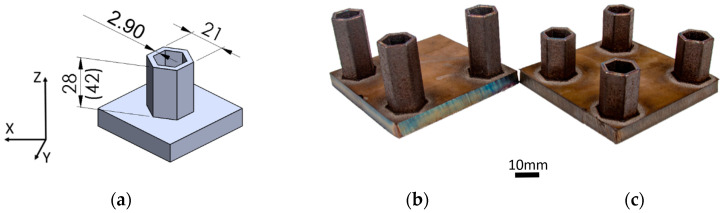
(**a**) CAD model of honeycomb design using SolidWorks (SHORT—height of 28 mm) with the dimensions of the build captured, (**b**) DED-processed sample—LONG (height of 42 mm) and (**c**) SHORT (height of 28 mm).

In this study, two honeycomb structures (Figure 1a) were designed with different heights to capture the influence of buckling and fracture modes under compressive loading. The shape of the part and loading conditions were selected based on an extensive study of the literature, and finally, inspired by Yang [21], Buchanan [22], and Anandan [23]. The structures were designed to be used as a support structure in general machinery and the nuclear and automotive industries. In this study, these DED-processed parts represent a quite complicated geometry featuring symmetry and sharp angles which were tested in as-deposited state without post-processing, i.e., milling. The structures were designed with height of 28 mm and 42 mm (Figure 1b,c) and called SHORT and LONG, respectively. The wall thickness of 2.9 mm was equal for both structures. It should be noted that it was a minimum value considering the possibilities of applied DED technology.

As mentioned above, the material model was created based on the results of the mechanical testing of miniaturized samples cut from the manufactured structures performed under quasi-static conditions at room temperature. It is noteworthy that the uniaxial tensile test (UT) specimens do not meet the condition of proportionality specified in the standard due to miniaturization. Furthermore, the samples for plane strain (PS), compression (CT), and biaxial test (BT) cover a wide range of stress states and were used to determine fracture locus. All samples were manufactured by a wire electric discharge machine (WEDM) cutting. The tensile test samples were tested with fully machined surface (with thickness reduction) and with the original surface in as-deposited state (without thickness reduction).

The determination of the material model was supported by detailed metallography observation of deposited structures including the hardness measurement. Based on the experimental results, the plasticity model was calibrated and the experimental-numerical approach was used to evaluate the loading path to fracture in order to determine the fracture strain, triaxiality, and Lode parameter to generate the fracture locus of the DED-processed honeycomb structure.

## 2. Material and Additive Manufacturing Process

### 2.1. Powder and Process

**Figure 2:**
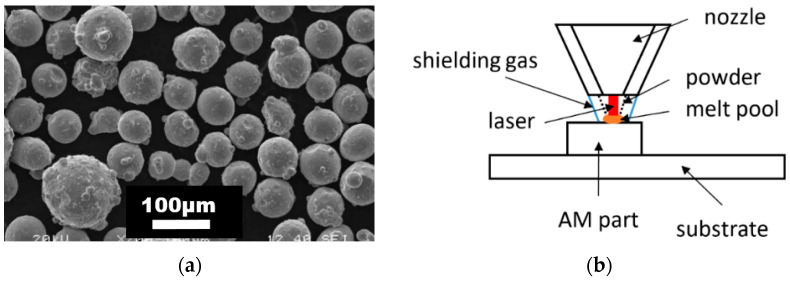
(**a**) SEM micrograph of the 316L powder feedstock with spherical particles of 53–150 µm diameter used in the production of honeycomb structures, (**b**) Schematic illustration of the blown powder DED process.

**Figure 3:**
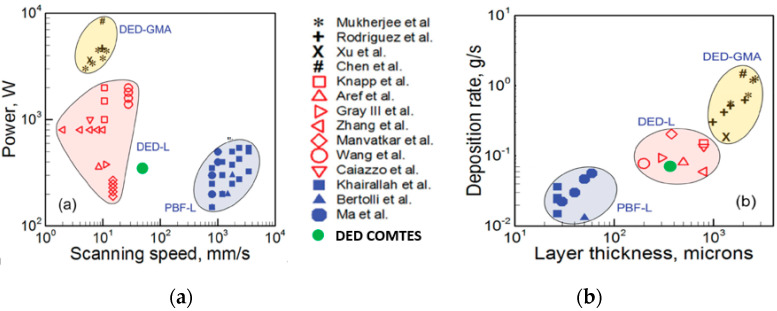
DED processing parameters summarized by Mukherjee et al. (**a**) power vs. scanning speed [32], (**b**) deposition rate vs. layer thickness [32]; the parameters applied in the current study were marked as green dot (DED COMTES).

The honeycomb structures were made of stainless steel 316L powder feedstock with spherical particles (see Figure 2a) with 53–150 µm size supplied by the commercial supplier Sandvik Osprey Ltd. (Wales, UK). The chemical composition of both, the powder feedstock and substrate, is summarized in Table 1. The deposition system employed in this study was an InssTek MX600 Blown Powder DED machine (InssTek, Daejeon, Korea) equipped with a 2 kW Ytterbium fiber laser. The process is schematically illustrated in Figure 2b. Figure 3 presents the DED deposition parameters summarized by Mukherjee [32]. The green points refer to the processing parameters applied in this study (DED COMTES). At the defined mean value of power, a higher scanning speed was obtained in comparison to the above-mentioned literature data.

**Figure 4:**
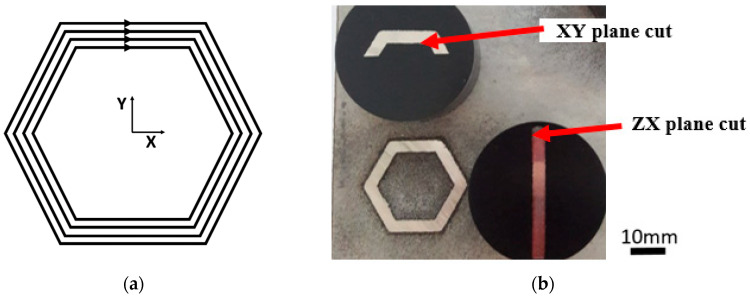
(**a**) The scanning strategy of the DED-processed honeycomb structures—four scans over the thickness of the component, (**b**) The samples embedded in the resin for metallographic observation extracted in two parallel planes (XY- and ZX-oriented).

A base plate of 100 mm × 100 mm × 10 mm made of commercially available 316L was utilized in the experiment. The dimensions are given by a holder of the DED machine. In order to remove potential residual stresses, the base plate was annealed (400 °C for 4 h) before deposition. The scanning strategy and scanning direction for the initial layer are schematically shown in Figure 4a. The starting position of the following welds was rotated by 90° between layers and the scanning direction was opposite. The structure consisted of four scans over the wall thickness. The thickness of the layer was 0.25 mm. The laser power with the mean value of 400 W was driven by the Direct Metal Tooling mode (DMT mode). The DMT mode automatically controls the laser output according to the thickness of the layer. The powder was scanned with speed of 14 mm/s. Argon gas with a purity of 4.8 was used as a protective atmosphere. The building process took 1.75 and 2.5 h for the SHORT and LONG sample, respectively.

The honeycomb structures were subjected to neither thermal treatments nor machining. The surface was left as it was fabricated, where the surface roughness of Ra ~9 µm was recorded.

### 2.2. Metallography Investigations and Hardness Measurement

**Figure 5:**
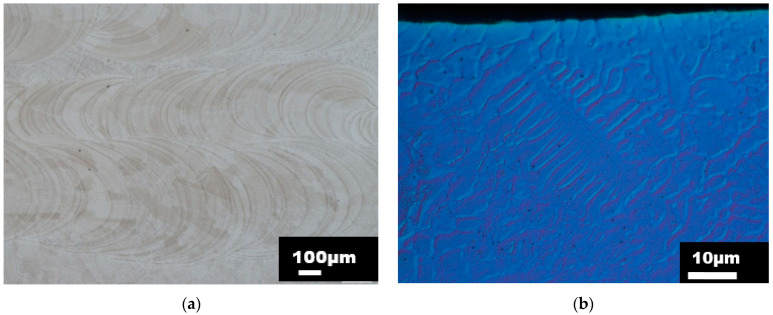
(**a**) Light micrograph of a melt pool path captured in XY plane (perpendicular to build direction), (**b**) Light micrograph with differential interference contrast (DIC) captured in XY plane (higher magnification).

**Figure 6:**
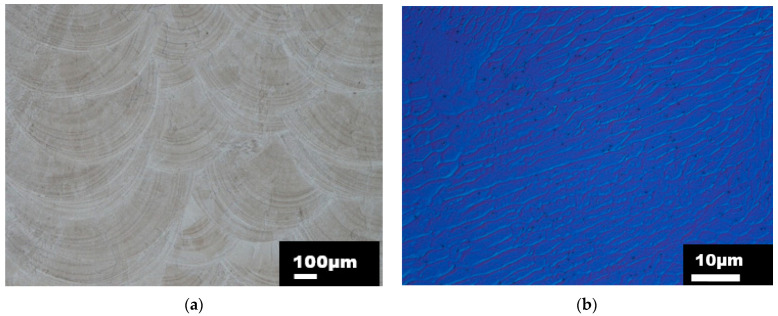
(**a**) Light micrograph of a melt pool path captured in ZX plane (parallel to build direction), (**b**) Light micrograph with differential interference contrast (DIC) captured in ZX plane (higher magnification).

The sample dedicated for metallographic observations was cut in the XY plane (perpendicular to the build direction) and in the ZX plane (along the build direction) as shown in Figure 4b. The samples and cutting planes were designated following the document ASTM WK49229. Prior to examination, the samples were subjected to a standard metallographic preparation, including etching with a V2A chemical agent. The sample was investigated using a NIKON ECLIPSE MA200 light microscope, which is equipped with NIS Elements 5.2 software. The metallographic analysis proved the occurrence of a typical line-shaped morphology denoting melt pools. The examples of the melt pools and microstructure details are depicted in Figure 5a,b for the XY plane and Figure 6a,b for a ZX plane.

In both melt pool path visualizations (Figure 5a and Figure 6a), the size of the melt pools varied due to the use of DMT mode and the associated change of the laser power. The microstructure was formed by an austenitic matrix with a fine cell substructure, columnar grains (ZX plane) with evenly distributed globular pores. This type of microstructure, which is similar to the directional solidification of the microstructure, is the result of epitaxial and dendritic grain growth in a direction identical to the direction of the heat flow. In the surface areas of the material, the grains acquired the preferred orientation towards the center of the DED-manufactured structure, which is related to heat dissipation and temperature gradient. The sub-surface grains (observed in the ZX plane) also reached narrower dimensions in comparison to the rest of the material. The average grain size established on the XY plane was approximately 75 µm.

**Figure 7:**
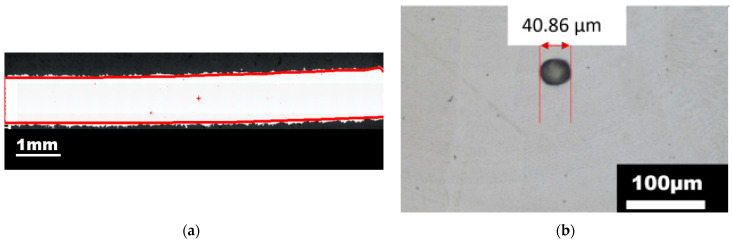
(**a**) The evaluation area of porosity analysis—ZY plane (parallel to build direction), (**b**) Results of porosity analysis—pore of the largest diameter (40.86 µm) found in the structure observed using light microscopy.

The porosity was evaluated in the ZX cut plane and the maximum size of the pore found in the microstructure was 40.7 µm (see Figure 7a). The total porosity was evaluated as 0.013%, as shown in Figure 7b. From the metallographic point of view, the detected presence of pores did not have a significant effect on the microstructure and material failure. This hypothesis is consistent with other studies of Komischke [34] and Wilson-Heid [35]. Based on the evaluation of previous studies, Komischke [34] stated that the material local separation starts when the size of non-homogeneity is larger than 50 µm. Wilson-Heid [35], in his study of the L-PBF-processed 316L steel, found that for the tensile test results the influence of pores can be spotted when they are larger than 180 µm.

**Figure 8:**
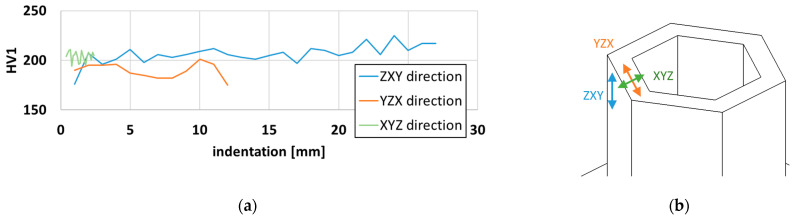
(**a**) Results of hardness measurement—HV1 distribution of hardness with measurement step of 1 mm along three directions, (**b**) schema of honeycomb structure with the directions of measurement ZXY, YZX and XYZ presented.

The hardness HV1 was evaluated by a laboratory hardness tester Struers Durascan 50 (EMCO-TEST Prüfmaschinen GmbH, Kuchl, Austria) with a normal force of 9.807 N. The measurement step was 1 mm. The measured hardness distribution for the ZXY, XYZ, and YZX directions is displayed in Figure 8. The ZXY direction hardness profile demonstrates that the hardness is continuously increasing with the deposition height from 175 HV to 225 HV. In the case of YZX direction, the hardness values lie between 175 HV and 201 HV. The observed oscillation of the values is probably due to the different positions of measurements within individual melt pool.

The metallographic investigations demonstrate the material homogeneity along the individual sections of the honeycomb structure. The presence of “non-critical” pores was also observed. Together with the constant hardness profile in individual directions, the investigations proved to have no significant effect of the material’s microstructure on the material failure, which implies the possibility of using an isotropic model.

## 3. Computational Models

Many material models describing ductile fracture have been developed and published either in the strain or stress space or their combination. The metallographic investigation revealed no effect of the microstructure on the material failure, so using the isotropic model was possible. The material models used in this paper are based on the classical incremental plastic response with isotropic hardening. The Von Mises yield rule and classical J2 theory were also applied based on Wang’s work [13], as with our previous study with Azinpour [18].

Based on the evaluation in our previous study [36], the phenomenological concept of damage in continuum mechanics, which was described using Von Mises stress q, stress triaxiality η, and Lode parameter ξ was applied. These quantities are defined using the second and third invariant of deviatoric stress according to Equation (1).

(1) $$J_{2}=1/2\left(S_{1}^{2}\;+\;S_{2}^{2}\;+S_{3}^{2}\;\right)J_{3}=S_{1}S_{2}S_{3}$$

Principal deviatoric stresses $S_{1}$, $S_{2}$ and $S_{3}$ are principal values of stress deviator:

(2) S=σ+pI

where *p* is hydrostatic stress and it is defined by Equation (3):

(3) p=−1/3 tr(σ)

Von Mises stress *q* is defined in Equation (4):

(4) $$q=\sqrt{3J_{2}}$$

Stress triaxiality *η* can be expressed by Equation (5):

(5) η=−p/q

The Lode parameter *ξ* can be expressed by the following Equation (6), where the third invariant was included:

(6) $$\xi =\frac{27}{2}\frac{J_{3}}{q}$$

The normalized Lode angle can be expressed according to Equation (7) and is called the Lode angle parameter:

(7) $$\bar{\theta }=6\theta /\pi =1-2/\pi \arccos\xi$$

The model of plastic response works with the simple surface of plasticity that is based on Von Mises stress according to Equation (8):

(8) $$q=\sigma_{Y}\left({\overset{¯}{\varepsilon }}_{pl}\right)$$

An associated flow rule has only one history-dependent state parameter—the accumulated intensity of plastic strain, referring to accumulated plastic strain expressed by Equation (9):

(9) $${\bar{\varepsilon }}_{pl}=\int {\dot{\bar{\varepsilon }}}_{pl}dt$$

where $\dot{\;{\bar{\varepsilon }}_{pl}}$ is an equivalent intensity of plastic strain:

(10) $${\dot{\bar{\varepsilon }}}_{pl}=\sqrt{\frac{2}{3}{\dot{\varepsilon }}_{pl}:{\dot{\varepsilon }}_{pl}}$$

The relation $\sigma_{Y}\left({\overset{¯}{\varepsilon }}_{pl}\right)$ is calibrated experimentally. The failure criterion is based on phenomenological quantity damage D that is defined as a non-decreasing scalar parameter (Equation (11)) that depends on loading history and can be understood as the linear accumulation of incremental damage in the process of monotonic loading

(11) $$D=\int_{0}^{t}\frac{{\dot{\bar{\varepsilon }}}_{pl}\;dt}{{\bar{\varepsilon }}_{f}\left(\eta ,\bar{\theta }\right)}$$

**Figure 9:**
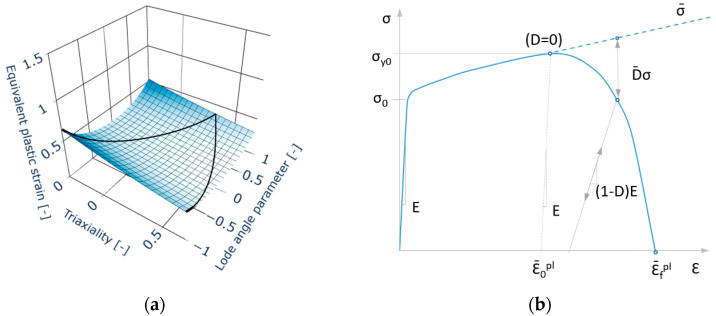
(**a**) Asymmetric fracture locus proposed by Wierzbicki [37], (**b**) Engineering stress-strain curve with description of parameters applied in numerical simulation.

The fracture locus $\bar{\varepsilon_{f}}$ is a parabolic function of stress triaxiality η and the Lode angle parameter $\bar{\theta }$. The influence of stress triaxiality is described in exponential form and has to be calibrated experimentally. The ductile fracture of the material occurs as soon as critical damage value *D_crit_* is reached. The fracture locus has the physical meaning of accumulated plastic strain at the instant of ductile damage initiation at the end of hypothetic monotonic loading with constant both, triaxiality and the Lode angle parameter. In such a loading process, the damage at failure reaches the value *D_crit_* = 1. More information can be found in the work of Wierzbicki [37], Bai [15], Chai [38] and Bai [39], who expected the fracture locus to be asymmetric, Figure 9a. This model is known as Modified Mohr-Coulomb (MMC), see Beese [40]. Fracture locus was calibrated based on the previous study [41].

The MMC is expressed in Equation (12). The material constants *c*_1_, *c*_2_, and *c*_3_ are obtained by a calibration procedure based on the results of experimental measurement.

(12) $${\bar{\varepsilon }}_{f}={\left\{\frac{A}{C_{2}}\left[C_{3}+\frac{\sqrt{3}}{2-\sqrt{3}}\left(1-C_{3}\right)\left(\sec\left(\frac{\bar{\theta }\pi }{6}\right)-1\right)\right]\left[\sqrt{\frac{1+C_{1}^{2}}{3}}\cos\left(\frac{\bar{\theta }\pi }{6}\right)+c_{1}\left(\eta +\frac{1}{3}\sin\left(\frac{\bar{\theta }\pi }{6}\right)\right)\right]\right\}}^{-\frac{1}{2}}$$

The constants *A* and *n* originate from the simple power hardening law of the Swift model (Equation (13)) representing one–to–one mapping by a power law expression in the elastic-plastic region.

(13) $$\bar{\sigma }=A{\bar{\varepsilon }}^{n}$$

The artificial degradation function described by a parameter of degradation D is implemented in Abaqus software [38] in order to prevent the gradual loss of stiffness in the whole element when equivalent plastic strain is at the onset of damage (D = 0). The degradation is not included as a material parameter and, therefore, it is not included in the calibration process. Nevertheless, the results of the numerical simulation can be affected by the degradation. Since the failure is indicated in the element of Finite Element (FE) mesh, the degradation manifesting itself as a decrease of elastic modulus is triggered according to Equation (14). Once the critical value *D_crit_* = 1 is reached in the element, this element is removed from the FE mesh. The process starting with fracture initiation *ε_finit_* over damage softening up to the final fracture can be seen in Figure 9b.

(14) $$E^{*}=\left(1-D\right)E$$

## 4. Small Samples Design, Testing Device, and Strain Measurement

**Figure 10:**
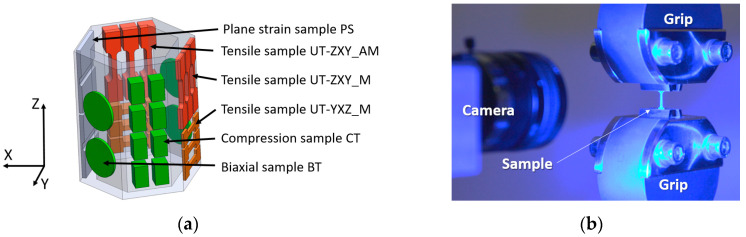
(**a**) Cutting plan of DED-processed honeycomb structure with uniaxial, plane strain and biaxial tension and compression specimen geometries included (**b**) Miniaturized tensile testing setup with miniaturized sample gripped and optical (DIC) deformation tracking.

The material properties of the honeycomb structure were measured using miniaturized samples, which were machined according to the cutting plan presented using SolidWorks in Figure 10a. Table 2 shows the sample portfolio tested within the experiment. The miniaturized tensile test (MTT) samples were employed, since standard tensile test samples could not reliably assess the local material differences as stated by Džugan [39]. Moura et al. reported that the application of a miniaturized tensile specimen is reasonable when an insufficient amount of material is available or to reduce the costs of specimen production and testing. Nevertheless, the selection of the specimen geometry is crucial since the slimness ratio *k*, which is the relation between initial gauge length and specimen cross-sectional area, directly affects the elongation value [42]. The usability of the MTT technique to assess the mechanical properties of various structure states was previously demonstrated for conventionally and additively processed materials by Chvostova et al. [40], Gotterbarm et al., and Džugan et al. [43]. The application of MTT specimens was also presented by Melzer et al. In this study, the sub-sized specimens were employed in order to investigate local mechanical properties of the interface of two different materials in a functionally graded block providing a deep insight into the location- and orientation-related properties [44].

The MTT was performed following the internal methodology RD 2/30 (accredited) according to the standard DIN EN ISO 6892-1/ASTM E8 [42]. All tensile tests (uniaxial and plane strain, Table 2, rows 1–5) were performed using an electromechanical universal testing machine LabControl with a load cell of 5 kN capacity. Due to the small dimensions of the specimens, the strain measurement was carried out using the DIC system. The stochastic pattern was applied on the surface of the samples and the tests were recorded using a single camera (2D mode). The MTT setup is depicted in Figure 10b.

A biaxial test of small discs (Table 2, row 6) was carried out using a servo-hydraulic testing machine MTS with a load capacity of 10 kN utilizing special die equipment designed by COMTES. A quenched ball of diameter 2.37 mm was used as a punch. The punch displacement was measured using an axial mechanical extensometer clipped on the outer diameter of two parts of the die.

The compression test samples (Table 2, row 7) were tested using a Zwick Roell universal testing machine with a load cell capacity of 250 kN. The upper and lower platens were lubricated by copper grease to reduce the friction between them and the samples. A cross-head movement was recorded by a single camera and its displacement evaluated using an optical system Mercury RT. The tests were performed at room temperature and cross-head velocity of 0.5 mm/min. At least three successful tests were performed for each geometry and orientation.

**Figure 11:**
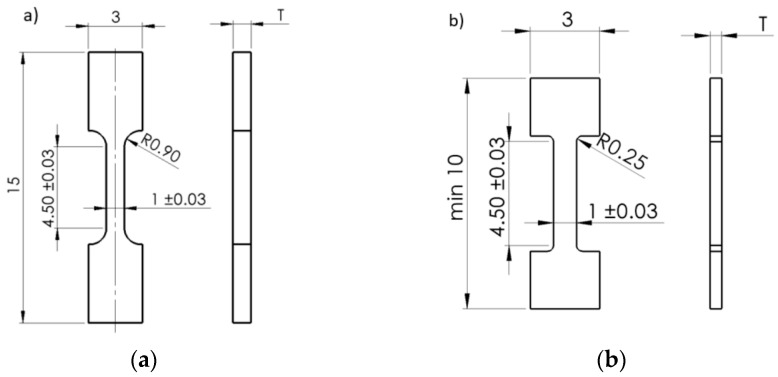
Geometries in millimeter of miniaturized tensile test samples (UT) in machined state applied in order to investigate the tensile properties of DED-processed honeycomb structures along (**a**) ZXY direction and (**b**) YZX direction designed using SolidWorks software.

The micro tensile test samples were extracted in two different orientations, ZXY and YZX. Two different sample geometries were applied due to the dimensions of the honeycomb structure. The shorter length of the shoulders and smaller radius of curvature of the neck for XYZ direction were proposed since the limited width of the honeycomb wall was available for testing. However, both MTT sample geometries can provide the same gauge length (Lo = 4 mm). The study aimed to describe the thin-walled structure behaviour; therefore, an application of the non-proportional MTT samples of the thickness corresponding to the component thickness appeared to be appropriate. In Figure 11, the dimension T stands either for thickness of the sample with original as-deposited surface (AM, approximately 2.90 mm) or fully machined samples (M, 0.50 mm).

**Figure 12:**
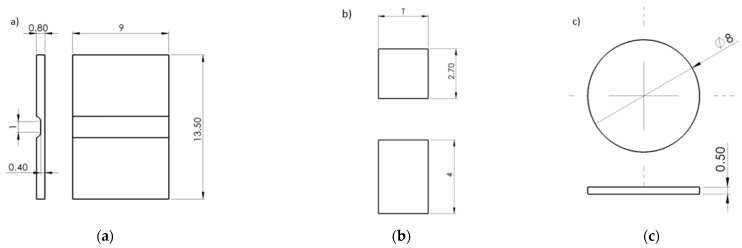
Geometries in millimeter of the samples designed in SolidWorks software extracted from DED-processed honeycomb structures employed in (**a**) plane strain test (PS), (**b**) compression test (CT) and (**c**) biaxial test (BT).

The plane strain specimens were fabricated in orientation ZXY with all surfaces being machined. The biaxial test samples were fully machined according to the drawing presented in Figure 12.

**Figure 13:**
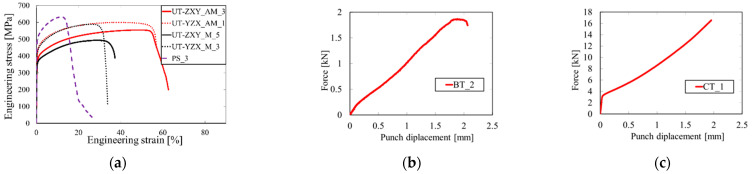
(**a**) Engineering stress-strain curves of different sample geometries and orientations, (**b**) force-displacement curve of the biaxial test, (**c**) force-displacement curve of the compression test.

The sample dimensions were measured before (a_0_, b_0_) and after the test (a_u_, b_u_). The tensile characteristics were evaluated, namely, yield strength (YS) and ultimate tensile strength (UTS). The results of the evaluated tensile parameters are summarized in Table 3. The representative engineering stress-strain curves for each sample type and orientation are depicted in Figure 13a. The force-displacement curves for the biaxial and compression tests are presented in Figure 13b,c, respectively.

## 5. Material Model Calibration

The material characterization including plasticity and damage was performed on test samples described in the previous chapter. The selected representative results of experimental measurement was subsequently used for the evaluation of fracture initization *ε_finit_*, triaxiality *η*, and the Lode angle parameter $\bar{\theta }$ by FEM simulation. All numerical simulations of mechanical tests have been performed with a standard version of Abaqus 2020. The explicit solver was used due to the possibility to include the damage and fracture. In addition, the C3D8R elements were applied in the evaluation. The actual dimensions of each sample were used according to Table 3.

### 5.1. Plasticity Model Calibration

**Figure 14:**
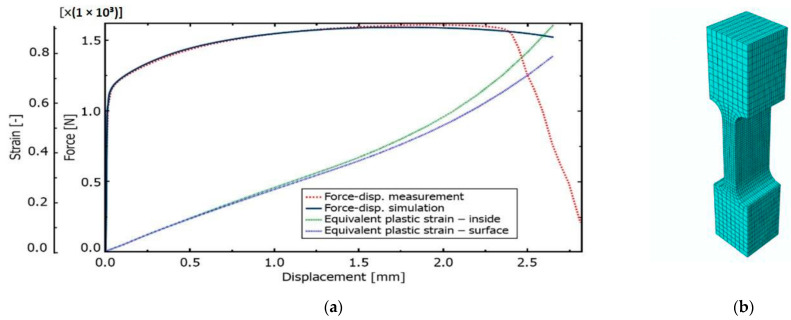
(**a**) Comparison of the measured and simulated force-displacement curve for ZXY_AM and FEM prediction of the relation between equivalent plastic strain inside the sample and on the surface (**b**) Finite element mesh in ZXY_AM sample (non-proportional with original surface).

The tensile test results of the sample UT-ZXY_M (as-deposited) were used as a reference. The plasticity including hardening was calibrated based on the measured force-displacement values for ZXY direction. The approach for determining initial uniaxial true stress-strain after necking was based on a weighted average method presented by Ling [43]. The comparison between the final hardening curve and experimentally measured data can be seen in Figure 14. Furthermore, the equivalent plastic strain (EPS) for critical elements in the middle of the sample thickness (INT) and on the surface of the sample (SURF) was determined based on a comparison of force-displacement drop and EPS trends indicating the ductile damage initiation.

### 5.2. Ductile Damage Model Calibration

The ductile damage initiation for the tensile test sample UT-ZXY_M was evaluated as *ε_finit_* = 0.7, which can be seen in Figure 14. The average triaxiality and Lode angle parameter were calculated as 0.32 and 1.0, respectively. The simulation using these values and the artificial degradation function is described by the parameter of degradation which is implemented in Abaqus as displacement at failure. The average fracture strain from numerical simulation εfinit is comparable with the value obtained experimentally based on the results of metallographic fracture surface measurements *ε_fract_*. The fracture strain based on the tensile test can be defined by Equation (15).

(15) $$\varepsilon_{fract}=\ln\left(\frac{A_{0}}{A}\right)$$

where *A*_0_ and *A* are the initial and final cross-section after a fracture, respectively. The surface area A was measured by image analysis using NIS-Elements digital image processing and analysis software. The values obtained based on Equation (15) are summarized in Table 4. This table consists of sample identification, comparison of the measured and simulated force-displacement curves, FEM simulation of the fractured sample, mesh size in the measured region, evaluated values of fracture initiation *ε_finit_*, adequate values of triaxiality *η*, Lode angle parameter $\bar{\theta }$ and additional information, such as the evaluation of *ε_fract_* based on Equation (15) for the tensile test, FEM model of testing setup for the biaxial test, and length of crack for compression test.

Based on the above-mentioned assumption concerning the isotropy, the hardening curve of as-deposited specimen, manufactured in ZXY orientation, was used for all samples and orientations including damage. There is a maximum discrepancy of 8% between the curves, but the overall mechanical response appears to be comparable, thus, the anisotropy definition was excluded from the study. The fracture strain EPS of YZX direction is identical to the value obtained in a vertical direction ZXY. Furthermore, the mechanical behaviour is comparable between the measurement and numerical simulation for both the vertical and horizontal directions. Regarding EPS evaluation, only the surface value was displayed, as this value is quite homogeneous through the sample thickness. The fracture strain based on the tensile test results was evaluated using Equation (15). However, it has to be considered that such an evaluation is strongly influenced by the mesh size in the critical region.

The plane strain test was used for further definition of the fracture surface since the plane strain condition was chosen to represent another stress state. The EPS for critical elements was determined based on a comparison of force-displacement drop and EPS trends indicating the ductile damage initiation. The comparison of simulation and measured values of force-displacement curves did not fully fit, therefore, the value of *ε_finit_* with a lower weighting factor was applied. It should be noted that the weighting factor represents the importance of the performed test in a testing portfolio.

The biaxial test was used to describe the equi-biaxial tension state with fracture, and this state defines the left boundary of fracture locus, which is discussed in the following paragraphs.

**Figure 15:**
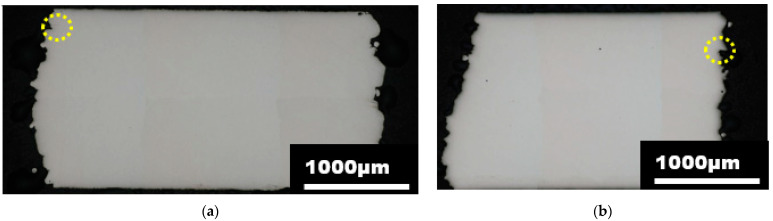
Cross-section (parallel to build direction) of the compression test sample—fracture creation on the surface of the compressed cube at displacement of 2 mm. (**a**) front view, (**b**) side view.

The fracture initiation of the uniaxial compression test was measured incrementally. To capture the value of the plastic strain fracture initiation, the interrupted tests with an increment of 0.25 mm starting at 1.0 mm, including metallographic observation of cut samples, were used. At a displacement of 1.5 mm, there was no fracture initiation. The fracture occured at 1.75 mm, and its length was 33.4 μm. A quite pronounced fracture appeared at 2 mm displacement and its length was 107.2 μm, as the detail can be seen in Table 4. The position of the crack is shown in Figure 15.

The basic four test sample shapes represent different stress states: the tensile test (UT/UT-ZXY), plane strain (PS), biaxial test (BT), and compression test (CT). The experiment has been performed using the ARAMIS DIC system to get strain on the sample surface to be compared with FEM simulation. The FEM simulation was used to get a representation of the force-displacement curve, strain distribution, and stress distribution. The equivalent plastic strain was used for finding the value of fracture initiation and adequate values of triaxiality *η* and the Lode angle parameter $\bar{\theta }$. Triaxiality and the Lode angle parameter represent the stress state, as was explained in the theoretical part of chapter 3.

**Figure 16:**
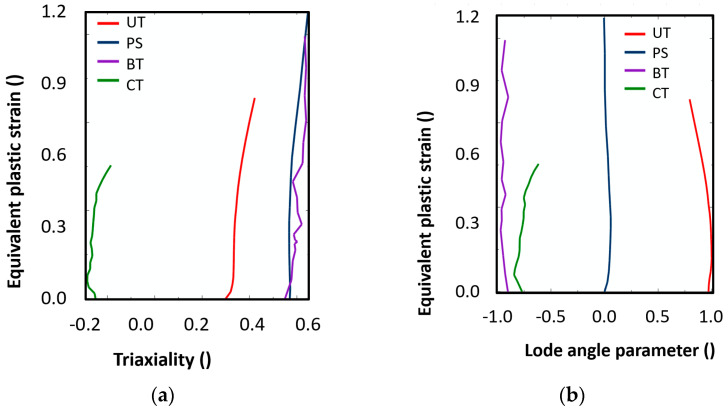
The curves representing dependence of (**a**) strain-triaxiality and (**b**) strain-Lode angle parameter for the samples under uniaxial, plane strain and biaxial tension and uniaxial compression.

The curves representing the triaxiality and the Lode angle parameter are displayed in Figure 16. The average values of stress triaxiality $\eta_{av}$ are calculated according to the following expression in Equation (16).

(16) $$\eta_{av}=\frac{1}{{\bar{\varepsilon }}_{f}}\int_{0}^{{\bar{\varepsilon }}_{f}}\eta \left({\bar{\varepsilon }}_{pl}\right)\;d{\bar{\varepsilon }}_{pl}$$

Lode angle parameter weighted average ${\bar{\theta }}_{av}$ is expressed in Equation (17).

(17) $${\bar{\theta }}_{av}=\frac{1}{{\bar{\varepsilon }}_{f}}\int_{0}^{{\bar{\varepsilon }}_{f}}\bar{\theta }\left({\bar{\varepsilon }}_{pl}\right)d{\bar{\varepsilon }}_{pl}$$

**Figure 17:**
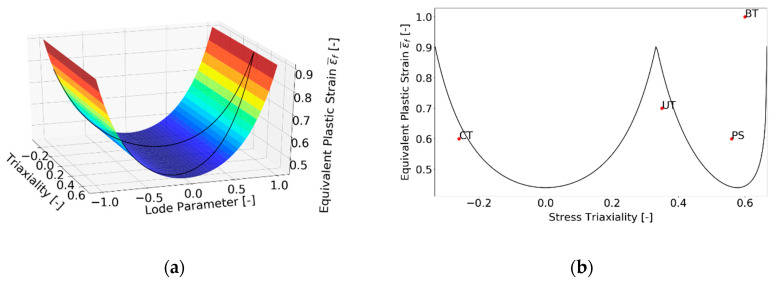
Fracture locus defined by MMC model of honeycomb (**a**) in Haigh-Westergaard space, (**b**) for plane stress condition.

The highest strain values were evaluated as initial fracture strain εfinit. The parameters (*ε_finit_*, *η*, $\bar{\theta }$), which were subsequently used for fracture surface evaluation, are summarized in Table 4. The experimental measurements were aimed to find a relation between the stress state and strain at the initiation and final fracture. In order to obtain three parameters *c*_1_, *c*_2_, *c*_3_ for the MMC criterion and fracture locus evaluation, at least three experiments representing distinct stress states were performed. Each set of values (*ε_finit_*,$\;\bar{\theta }$) specified in Table 4 represent a point in a three-dimensional space, through which the calibrated surface should pass with a specified approximation based on the minimization algorithm according to Equation (18). There are [*c*_1_, *c*_2_, *c*_3_] values representing the coefficients of the fracture locus of MMC. The software developed by COMTES using python scripts was applied to evaluate the MMC model in the modified Haigh-Westergaard space and for plane stress condition as can be seen in Figure 17.

(18) $$\left[c_{1},c_{2},c_{3}\right]=MIN\;\left[\sum_{1}^{N}{\left(1-D_{i}\left(\eta ,\bar{\theta },{\overset{¯}{\varepsilon }}_{f}\right)\right)}^{2}\right]$$

The well-documented steps for fracture locus construction as published by Bai, Wierzbicki [45], Mohr [16,46], and Lian, Munstermann [47] were followed. The calibrated material constants of Swift and MMC models are shown in Table 5.

**Figure 18:**
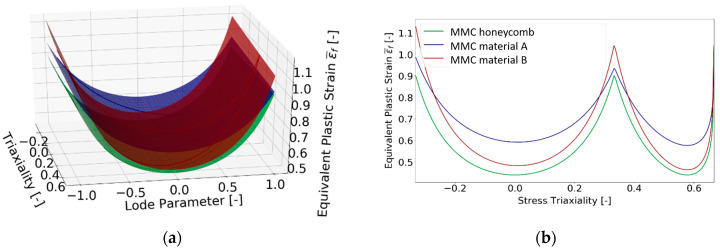
Comparison of MMC model for material 316L from different suppliers (material A—blue color, material B—red color) [48] and honeycomb (current study—green color) (**a**) in Haigh-Westergaard space, (**b**) for plane stress condition.

The fracture surface derived in this study was compared with the one published by Paredes et al. [48], where the mechanical tests of two conventionally produced austenitic steels 316L from different suppliers A and B were performed. For the fracture surface, they used the MMC model and all the parameters were published, namely the equivalent plastic strain to fracture, stress triaxiality and normalized Lode angle. The MMC models in the modified Haigh-Westergaard space and for plane stress condition for honeycomb structure (current study, green curve) and two materials from supplier A (Paredes, blue curve) and supplier B (Paredes, red curve) are depicted in Figure 18. The material was assessed as isotropic, and mechanical properties were determined in the rolling direction.

## 6. Honeycomb Experimental Compression Test

**Figure 19:**
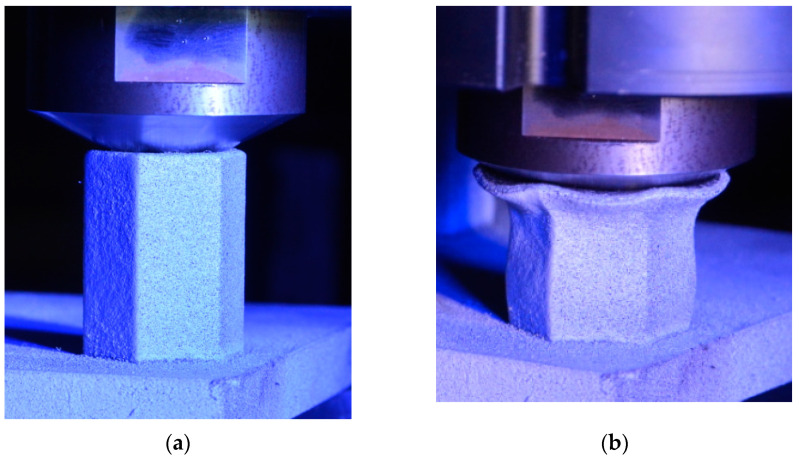
Setup of compression test of the honeycomb structure with a stochastic pattern for deformation tracking using DIC (**a**) before the test, (**b**) after the test.

In order to assess the mechanical performance of the manufactured honeycombs, the compression test was employed. The punch was characterized by a conical shape with an angle of 126°. Two different honeycomb structures with a height of 28 mm (SHORT) and 42 mm (LONG) were tested. In order to reduce friction, the punch surface was polished. No lubricant was applied between the punch and honeycomb structure. The tests were performed using a Zwick Roell universal mechanical testing machine with a maximum load capacity of 250 kN. The tests were performed at room temperature with a cross-head speed of 1 mm/min. All samples were recorded by means of an Aramis DIC system by GOM using two 12 MPx cameras (3D). The test setup before the test and at the end of the test is depicted in Figure 19a,b, respectively.

**Figure 20:**
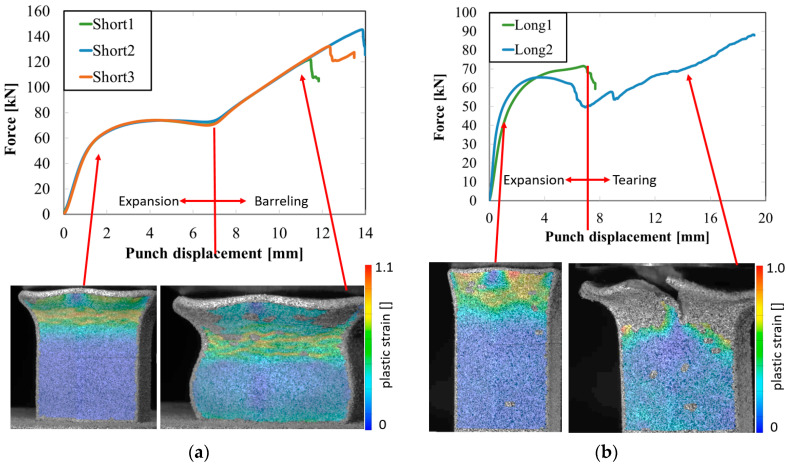
Records of honeycomb compression test: (**a**) force-punch displacement records for a SHORT honeycomb compression test, (**b**) force-punch displacement records for LONG honeycomb compression test.

The records of force-punch displacement for the SHORT honeycomb compression tests are shown in Figure 20a. The records can be split into two parts. At the beginning, the deformation of the honeycomb in terms of elastic behaviour was characterized by the linear dependency of force-punch displacement. As the punch displacement continued, the deformation began to apply through the expansion of the upper part of the honeycomb. On the record of force-punch displacement, it could be observed that the corresponding part of the curve is no longer linear. After the honeycomb reached a certain limit for further expansion, the additional deformation mode appeared, and distinct barreling of the lower part was observable. This moment was characteristic of a sudden increase of the loading force and linear dependency of force-punch displacement records. Although during the barreling stage the expansion was still visible, the barreling was the major deformation mode. For this reason, in Figure 20a this stage is called barreling only.

During the compression test, the deformation process of LONG honeycomb differed from the described SHORT honeycomb. The results of the compression test of LONG honeycomb are visualized in Figure 20b. At the very beginning, when the punch came into contact with the honeycomb, the deformation was identical to the deformation behaviour of SHORT honeycomb. Firstly, elastic loading and linear dependency of the force-punch displacement record were visible. At a certain moment, the expansion was a major deformation process. After the continuous loading, the material failure appeared within the expanded volume of the material in the upper part of the honeycomb. This is the main difference from the compression behaviour of the SHORT honeycomb, where distinct barreling appeared. As the compression test continued, the force level was increasing. However, no distinct barreling was observed in this case since considerable tearing continued.

The end of the expansion of SHORT and LONG honeycombs was noticed at the same punch displacement of 7 mm (Figure 20a,b). The reason could be the same sample cross-section; however, the sample behaviour under loading after the expansion was dependent on the sample height.

## 7. Fracture Surface Observation

**Figure 21:**
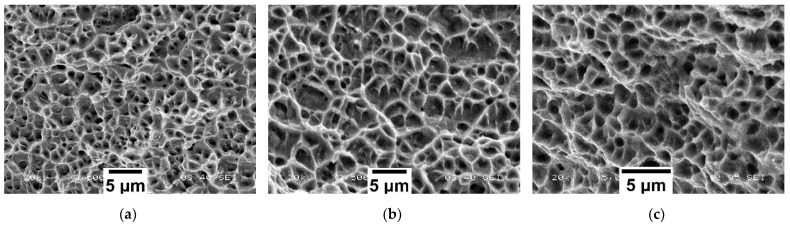
SEM micrographs of the fractured surface after (**a**) the tensile test, (**b**) compression test on SHORT honeycomb, (**c**) compression test on LONG honeycomb.

The mechanism of ductile fracture was observed on the fracture surfaces of the tensile test sample after the test and SHORT and LONG honeycomb structures after compression using a scanning electron microscope (SEM) JEOL IT 500 HR (JEOL Ltd., Tokyo, Japan). The fracture surfaces exhibited ductile fracture with dimple morphology and had a similar appearance for all samples. The dimples were equitable and no parabolic dimples were observed. The fractures were generated by normal tensile stress with no or minimal contribution of other stresses. There were a large amount of dimple initiation places since the fractures are formed by coalescence of a large number of fine dimples. Based on this observation, it can be concluded that the prevailing fracture mechanism in the honeycomb structure is a tension mode of tearing. The micrograph of the fracture surface of the tensile test sample, as well as SHORT and LONG honeycomb structures, are shown in Figure 21a–c, respectively.

## 8. Honeycomb Numerical Compression Test

**Figure 22:**
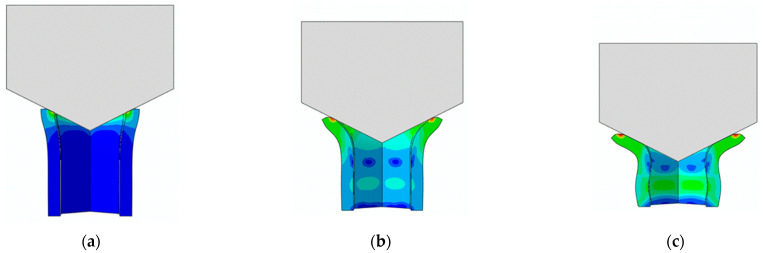
Behaviour of SHORT structure under compressive loading in time depicted using FEM simulation (**a**) the first contact between punch and sample, (**b**) sample expansion, (**c**) sample barreling.

The main goal of the honeycomb numerical compression test was to verify the material model created using the results of the experimental one. The calibrated material models were based on the uncoupled phenomenological ductile damage model, which consists of isotropic plasticity evaluation and the MMC model. An extensive study concerning the FE mesh quality was utilized. The final density used for all calculations was 0.8 mm × 0.5 mm with 7 elements through the wall thickness representing 192,060 elements in total for the honeycomb structure. The punch was modelled as a rigid body and the support plate was an elastic body. During the experiment, several phases of the structure compression test defined as expansion and barreling can be seen as shown in Figure 22.

### 8.1. Short Honeycomb Numerical Testing

**Figure 23:**
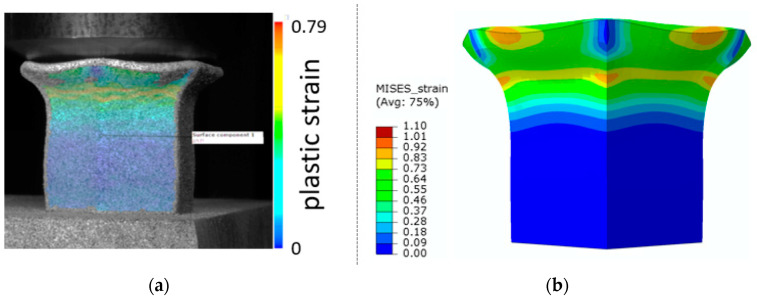
Comparison of SHORT structure behaviour at the end expansion phase using plastic strain distribution and total deformation evaluated by (**a**) mechanical test (**b**) FEM simulation.

The total equivalent strain was evaluated using ARAMIS software at the surface at the end of the compression test, which is depicted in Figure 23a for the experimental measurement. The total equivalent strain was plotted using the Abaqus field variables in Figure 23b. Both maximum equivalent plastic strain values and distribution can be compared in barreling phase, as specified in Figure 23.

Based on the results of the initial numerical test without damage, a friction coefficient (Coulomb law) equal to 0.1 in the expansion phase and 0.5 during the barreling phase are the most relevant for simulation of the whole compression process. This approximation is based on the surface roughness measurement as specified in chapter 2 and the results published by Parthasarathi [49]. The view on the upper part of the honeycomb structure is shown in Table 6 in the second line. The comparison of the measured and simulated force-displacement curves for the short structure with included damage is shown in Table 6. Regarding the force-displacement curve, the agreement is relatively good, nevertheless, concerning the structure behaviour, the symmetric fracture pattern was achieved which is opposite to the experimental measurement where the fracture was indicated at one place only.

### 8.2. Long Honeycomb Numerical Testing

The next tested structure was a LONG honeycomb. The numerical simulation precisely predicted the behaviour that was observed during experimental testing as there was barreling, which went directly from the expansion stadium to the tearing mode. There is good agreement between the simulation and the experimental part, as can be seen in Table 6.

## 9. Discussion

This study is devoted to the relationship between the created FE model and the results of experimental measurement performed on a real DED-fabricated honeycomb structure. The rough surface of the part in an as-deposited state could be machined smoothly by milling. Nevertheless, this would cost additional effort, time, and expense, and may not be necessary or even feasible for some geometries. The mechanical properties of conventionally produced materials are mostly viewed as independent on the geometry used for evaluation. A completely different situation can be observed for the additively manufactured parts, as presented in the current case study. This problem has been extensively discussed by Roach et al. [15]. The size-dependent and machining effects can strongly, even stochastically, influence the material properties and therefore also the material model. From this point of view, a deposition and testing of additional samples seems to be problematic and unreliable. The selection of the sample dimensions, the evaluation of the obtained data and the transferring of the results to the evaluation of the whole-component behaviour has to be done with great care. These aspects are crucial for thin-walled structures due to a low number of grains through the thickness and higher porosity in a small volume of material. Thus, the aim of this study was a comprehensive description of the mechanical behaviour of the honeycomb structures based on experimental data and the results of numerical simulation covering all the above-mentioned effects. As stated by Saboori et al. [9], the mechanical properties can be considered as one of the main indicators of the quality of AM processes.

In the current investigation, two different honeycomb structures (Figure 1a) were manufactured from 316L powder using a laser-based DED process. The structures were 28 mm and 42 mm high, resembling the honeycomb support structure commonly used in, for instance, crash absorption structures. The structures were deposited using DMT mode, where at the defined mean value of power, a higher scanning speed was obtained in comparison to the literature data (Figure 3). Based on the metallographic investigation results, it can be stated that the microstructure of the DED-fabricated parts was characterized by a very low porosity of 0.013% and can be assessed as highly homogeneous. The size of the largest pore found in the microstructure was 40.7 µm, which is below the critical value for local material separation (50 µm) established by Komischke [34]. Furthermore, the hardness measurement in three different orientations revealed comparable HV1 values (Figure 8), hence, the isotropic material model was considered and the Von Mises yield rule and classical J2 theory were applied.

In this study, the material model for the honeycomb structure was created. The experimental data were used to perform the numerical simulation and compare it with the results of mechanical measurement. In order to perform the material characterization including plasticity and damage, several tests on different sample shapes with or without full machining of the sample surface were performed.

The miniaturized tensile test (UT) was performed on the non-proportional samples with the two surfaces in an as-deposited state and proportional, fully machined samples. The results revealed that the type of chosen sample geometry has a direct impact on the measured tensile properties and evaluated material model. The non-proportional samples were distinguished by higher ultimate tensile strength in comparison to the machined, proportional samples (Table 3). The dependence of the strength values on specimen dimensions was reported by Mukherjee et al. [32]. The energy input and thermal conductivity determine the microstructure, which affects the mechanical properties [7]. In the numerical investigation, Mukherjee et al. revealed that the number of parallel scans influence the dimensions of melt pools and cooling rates, which means that the specimen width affects the microstructure and mechanical behavior [32]. The phenomenon of lower ultimate tensile strength for machined specimens could also be observed due to an insufficient number of grains along the thickness of machined specimens. In the previous studies of Chan and Fu [50,51], a dependence of the stress on the number of grains within the cross-sectional area of the sample was investigated. The results demonstrated that with a decreasing number of grains, the evaluated stress is decreasing. Based on the measured average grain size of the additively manufactured honeycomb, the number of grains within the UT sample cross-section was 40 and six for non-proportional and proportional samples, respectively. According to Konopik et al. [48], the minimum number of grains within the sample thickness is 10. Therefore, the data obtained from the tensile test performed on non-proportional samples were used in the numerical simulation, since this geometry meets the requirements related with the minimal number of grains along the sample thickness. The proportional samples manufactured in both orientations were excluded from fracture locus evaluation. ZXY_AM samples were selected for further evaluation since their orientation was consistent with the built direction (Z), the direction of extraction of mechanical test samples of other geometries and the loading conditions in the honeycomb compression test. Due to the maximum discrepancy of 8% between the hardening curves of both orientations, YZX_AM samples were not considered.

The material model used in this study was based on classical incremental plastic response with isotropic hardening and the phenomenological concept of damage in continuum mechanics. This model is described in the stress state in terms of Von Mises stress. The linear accumulation of incremental damage in a process of monotonic loading was applied. Fracture locus $\bar{\varepsilon_{f}}$ is a function of stress triaxiality and the Lode angle parameter. Both parameters were calibrated experimentally according to the previous study [50].

The hardening curve was calibrated according to the Swift model formula and the ductile damage initiation was evaluated based on tensile test results performed on the non-proportional sample. The fracture strain evaluation was supported by the results of the metallographic analysis. The calibrated hardening curve was used for simulation of plane strain, biaxial tension and uniaxial compression states (Table 4). A good agreement was obtained for the uniaxial tensile test for proportional samples as well as for the biaxial tension and uniaxial compression tests. Surprisingly, the results for the plane strain state were overestimated by numerical simulation, therefore the higher value of ductile damage initialization was calculated.

The evaluated damage model was compared to the damage model of conventionally processed 316L material published by Paredes [48]. The MMC damage model for plane stress conditions is visualized in Figure 18. The comparison demonstrates that the additively manufactured honeycomb structure made of 316L is characterized by lower values of equivalent strain to fracture in the entire range of stress triaxiality compared to the conventionally processed 316L.

The most important part of this study was the comparison of the behaviour of the derived material model in the simulation of two different structures with the experimental results. In order to reach this goal, the honeycomb structures were manufactured with various heights, but the same thicknesses. The ratio of heights between structures was 1:1.5 and for SHORT honeycomb structure, the ratio between thickness and height was 1:10. It could be expected that different structure heights cause different behaviour under loading in terms of structural stability and buckling.

The honeycomb compression test was used for the verification of calibrated material models. The behaviour of the structure under static loading was consistent with the results of FEM prediction. Regarding the force-displacement curve, the agreement was relatively good for both structure geometries. In the case of SHORT honeycomb, due to symmetric conditions set in the simulation, the crack was initialized in multiple locations, while the real fracture was indicated only in one place. The simulation was performed for two different friction coefficients. The friction coefficient of 0.5 was determined based on the roughness measurement of surfaces made by AM (Ra ~9 µm). The constant friction coefficient was not applicable for numerical simulation of the SHORT honeycomb structure, therefore a non-constant friction coefficient was used in the ratio of 0.1–0.5 dependent on contact pressure. In contrast, the overall mechanical behaviour including fracture of LONG honeycomb was fully consistent with the numerical simulation, where a constant friction coefficient of 0.5 was used.

At the end, the fractographic analysis was employed in order to investigate and compare the mechanism of void coalescence on the fracture surface of the tensile test sample and honeycomb structure. All evaluated fracture surfaces had the same character, i.e., dimples prevailing in tension mode of fracture. In the case of the honeycomb structure, the dominant mode of honeycomb fracture initiation indicated that the triaxiality range is rather positive with the low influence of the Lode angle parameter.

## 10. Conclusions

This study was focused on the prediction of the honeycomb structure behaviour under static compression loading with crack initialization using FE simulation. The mechanical behaviour under loading was evaluated using an experimental-numerical approach. The isotropic uncoupled material model was utilized, which was set up based on the results of mechanical testing of miniaturized samples extracted from the honeycomb structure. These samples of various geometries were used to describe different stress states. Subsequently, the fracture surfaces were investigated after the tensile and compression tests performed on the honeycomb structures. The triaxiality, Lode angle parameters, and fracture strain initialization were evaluated, and the Modified Mohr-Coulomb model was used for fracture surface description. The metallographic analysis revealed an austenitic matrix with a fine cell substructure, columnar grains, which grown epitaxially along the build direction Z and evenly distributed globular pores. The results of the hardness measurement and microstructure evaluation showed homogenous isotropic material properties of the honeycomb structure. This observation was confirmed by the results of mechanical tests performed using miniaturized tensile samples. Due to the isotropic behaviour of the material, the uncoupled material model was utilized, which was defined separately for the hardening curve and damage using the Swift model and Modified Mohr Columnb model, respectively. In the experimental part of the investigation, the proportional tensile test samples with reduced thickness were characterized by lower tensile strength that can be related to the lower number of grains along with the sample thickness. The calibrated material model, which was created based on basic mechanical test results, predicted the behaviour of the honeycomb structure under static loading. In the case of both geometries, the fracture was recorded at the same punch displacement value; however, the overall sample behaviour, i.e., tearing, was dependent on sample height. The fractographic analysis revealed that the prevailing fracture mechanism in the honeycomb structure is a tension mode of tearing.

## Figures and Tables

**Table 1:** Chemical composition of the powder feedstock and substrate used in the experiment (wt.%) [33].

Material Type	Cr	Ni	Mo	Mn	Si	Fe
Powder	17.2	10.4	2.3	1.3	0.8	Bal.
Substrate	16.2	10.5	2.1	1.1	0.4	Bal.

**Table 2:** Mechanical testing—sample portfolio including loading mode, surface state and thickness, orientation and designation of the samples.

	Strain State/Loading	Thickness	Surface	Designation	Orientation	Figure
1	Uniaxial tension/tensile test	0.50 mm	machined	UT-ZXY_M	ZXY	11a
2	Uniaxial tension/tensile test			UT-YZX_M	YZX	11b
3	Uniaxial tension/tensile test	2.90 mm	as-deposited	UT-ZXY_AM	ZXY	11a
4	Uniaxial tension/tensile test			UT-YZX_AM	YZX	11b
5	Plane strain/tensile test	0.38 mm	machined	PS	ZXY	12a
6	Biaxial tension/small punch test	0.50 mm		BT		12b
7	Uniaxial compression/compression test	2.90 mm	as-deposited	CT		12c

**Table 3:** Results of tensile, plane strain, biaxial and compression testing including the dimensions of the samples before and after testing, yield strength and ultimate tensile strength (if applicable).

Sample	a_0_	b_0_	a_u_	b_u_	YS	UTS
	mm	mm	mm	mm	MPa	MPa
UT-ZXY_AM_3	2.89/2.87	1.00	1.72	0.55	370	556
UT-YZX_AM_1	2.80	1.01	1.85	0.63	402	599
UT-ZXY_M_5	0.52/0.54	1.01	0.14	0.60	331	493
UT-YZX_M_3	0.63	1.01	0.17	0.56	384	518
PS_3	0.38	9.00	0.12	8.19	443	578
BT_2	0.52	-	-	-	-	-
CT_1	2.70	2.90	3.70	4.07	-	-

**Table 4:** Results of experimental and numerical investigation—comparison of stress-strain curves obtained by experimental measurement and simulation, equivalent plastic strain evaluation and metallographic fracture evaluation (if applicable) of the samples under uniaxial, plane strain and biaxial tension and uniaxial compression.

	Force—Displacement Curve	Equivalent Plastic Strain		Metallography Fracture Evaluation	
Uniaxial tension/tensile test	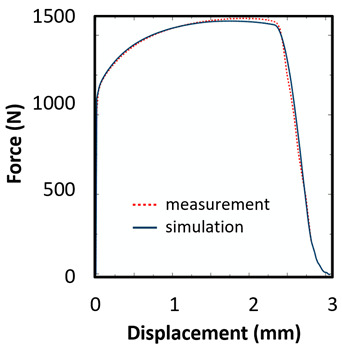	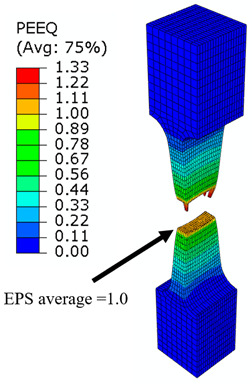	mesh 0.100 mm	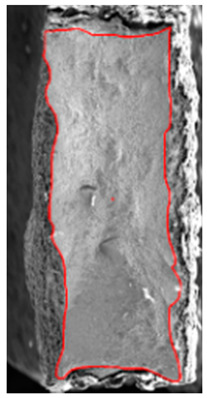	
			*ε_finit_* = 0.7		*A* = 2.89 mm^2^
			η = 0.35		*A_0_* = 1.17 mm^2^
UT-ZXY_AM			$\bar{\theta }$ = 0.95		*ε_fract_* = 0.90
			weight factor = 1.0		
Uniaxial tension/tensile test	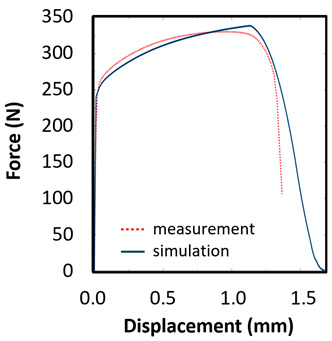	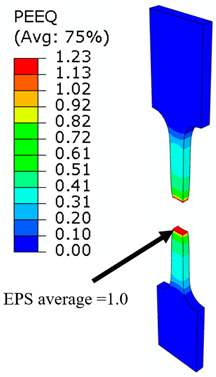	mesh 0.100 mm	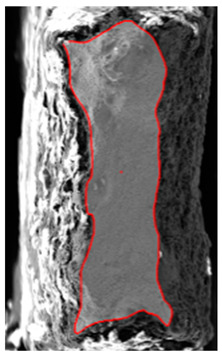	
			*ε_finit_* = 0.7		
			*η* = 0.35		A = 0.53 mm^2^
UT-ZXY_M			$\bar{\theta }$ = 0.95		A_0_ = 0.13 mm^2^
			weight factor = 1.0		*ε_fract_* = 1.37
Plane strain/tensile test	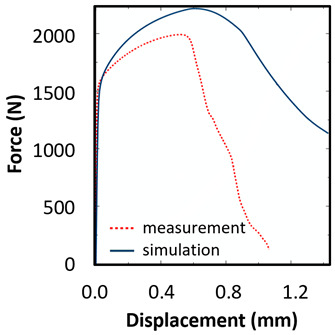	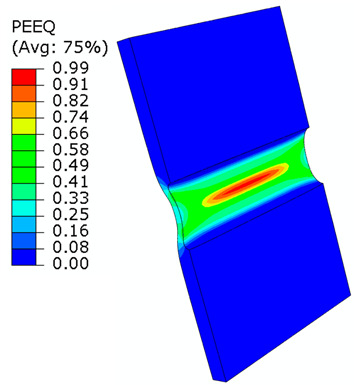	mesh 0.100 mm		
			*ε_finit_* = 0.6		
			*η* = 0.56		
PS			$\bar{\theta }$ = 0.04		
			weight factor = 0.5		
Biaxial tension/small punch test	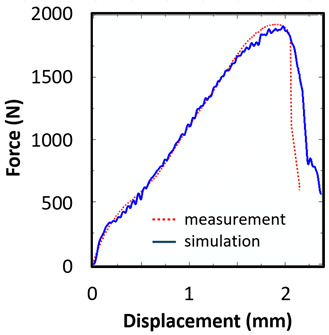	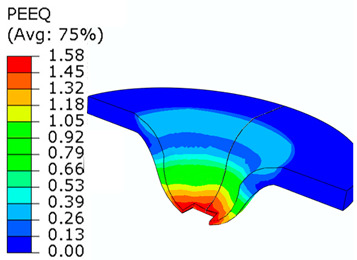	mesh 0.125 mm		
			*ε_finit_* = 1.0		
			*η* = 0.60		
BT			$\bar{\theta }$ = −0.96		
			weight factor = 1.0		
Uniaxial compression/compression test	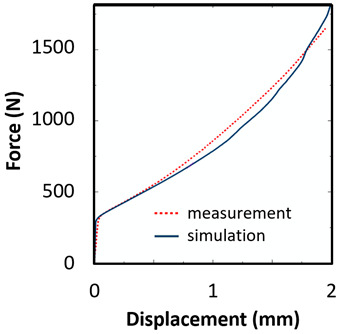	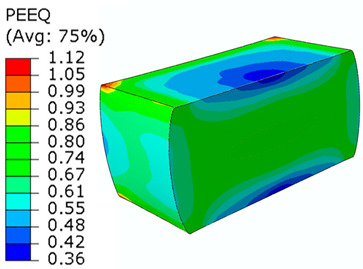	mesh 0.200 mm	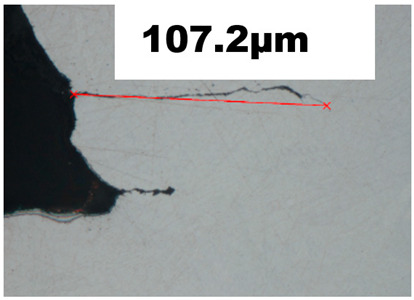	
			*ε_finit_* = 0.6		
			*η* = −0.26		
CT			$\bar{\theta }$ = −0.76		
			weight factor = 1.0		

**Table 5:** Calibrated parameters of Swift and MMC models.

Swift Model		MMC Model		
A	n	c_1_	c_2_	c_3_
1126 MPa	0.51	1.24 e^−13^	536	1.25

**Table 6:** This is a table. Tables should be placed in the main text near to the first time they are cited.

	Comparison of the Measured and Simulated Force-Displacement Curve	FEM Simulation	Compression Test
short	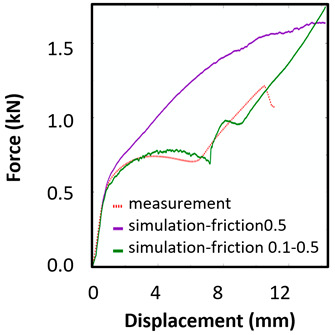	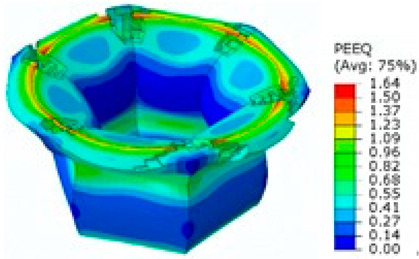	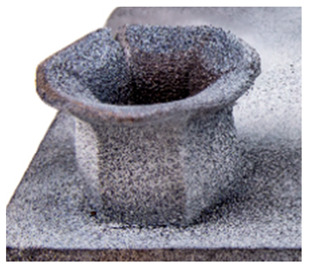
long	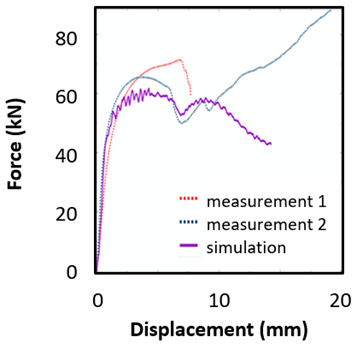	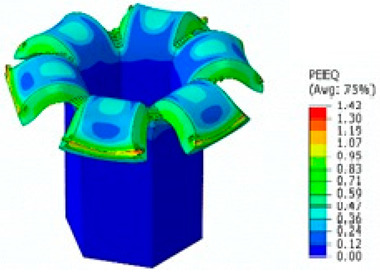	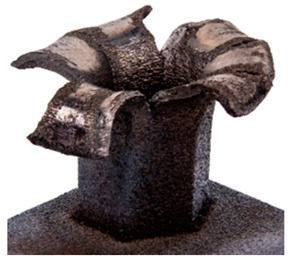

## Data Availability

Not applicable.
